# Risk factors for postoperative cognitive dysfunction in elderly patients undergoing surgery for oral malignancies

**DOI:** 10.1186/s13741-023-00330-2

**Published:** 2023-07-20

**Authors:** Yujia Wu, Cong Yu, Feng Gao

**Affiliations:** 1 Stomatology Hospital Affiliated Chongqing Medical University, Chongqing, China; 2 Chongqing Key Laboratory of Oral Diseases and Biomedical Sciences, Chongqing, China; 3 Chongqing Municipal Key Laboratory of Oral Biomedical Engineering of Higher Education, Chongqing, China; 4 The Sixth People’s Hospital of Chongqing, Chongqing, China

**Keywords:** Postoperative cognitive dysfunction, Oral and maxillofacial malignancies, Risk factors, Anaesthesia, Elderly patients

## Abstract

We aimed to analyse postoperative cognitive dysfunction (POCD) incidence and risk factors in elderly adults who underwent surgery for oral malignancies. A total of 112 elderly patients (aged ≥ 55 years) were selected for expanded resection of oral malignancy and cervical lymphatic dissection at our institution from December 2020 to December 2021. Participants were cognitively evaluated using the neuropsychological test scale 1 day before and 7 days after surgery to determine whether they had developed POCD. Based on whether POCD occurred 7 days after surgery, patients were classified into the POCD and non-POCD groups. Logistic regression was applied to perioperative factors to analyse the risk factors for POCD onset. Seven days after surgery for oral malignancy, there were 37 (33.1%) POCD morbidities. Multiple factor logistic regression analysis revealed that venerable age (odds ratio [OR] = 1.269, 95% confidence interval [CI] 1.056–1.525, *P* < 0.05), low education levels (OR = 0.792, 95% CI 0.644–0.974, *P* < 0.05), hypertension (OR = 4.153, 95% CI 1.335–12.732, *P* < 0.05), dyssomnia (OR = 1.272, 95% CI 1.001–1.617, *P* < 0.05), prolonged anaesthesia (OR = 1.009, 95% CI 1.001–1.018, *P* < 0.05), and intraoperative hypotension (OR = 5.512, 95% CI 1.240–24.506, *P* < 0.05) increased the POCD risk in elderly patients who underwent surgery for oral malignancies. Venerable age, low knowledge reserve, hypertension, dyssomnia, prolonged anaesthesia, and intraoperative hypotension are independent risk factors for POCD in elderly patients with oral malignancies.

## Introduction

Postoperative cognitive dysfunction (POCD) is a central nervous system complication that often occurs hours to days after surgery. Occasionally, onset can be postoperative months or even years, causing disorders in consciousness, cognition, orientation, memory, and sleep (Krenk et al. 2010). In the short term, POCD can aggravate other postoperative complications and increase the duration of hospitalisation and costs. In the long term, it has negative impact on recovery, reduces the life quality, and even increases the postoperative mortality ratio (Steinmetz et al. 2009). The global population is rapidly ageing, and the number of old individuals undergoing surgery has increased. Several clinical studies suggest a higher incidence of POCD in elderly patients at approximately 3–61% (Parra et al. 2011; Monk et al. 2008). Most POCD cases are reversible, but some patients are continuously affected. Some studies report that POCD still exists at 3 months after surgery and can progress from chronic disease to long-term cognitive impairment (Janoutova et al. 2015). Meanwhile, POCD has the same pathological manifestations as dementia (Alzheimer’s disease, AD). POCD can be considered a critical status between normal ageing and dementia, and 10–15% POCD transform to AD every year (Janoutova et al. 2015). At present, people are increasingly paying attention to perioperative brain health, and how to effectively prevent the occurrence of POCD is the top priority. Therefore, perioperative screening for risk factors in elderly patients to intervention treat in time can effectively prevent and treat POCD.

Over the past 30 years, the incidence and mortality of oral malignancies have increased annually (Fitzmaurice et al. 2017). According to a global survey, there are over 600,000 new cases of oral malignant tumours, accounting for approximately 10% of all cancers, making it one of the most common clinical malignant tumours (Torre et al. 2015). It affects the head, neck, and jaw, particularly the tongue, cheek, gums, jaw, salivary glands, and other important anatomical components, and can cause serious harm to human health (Marur and Forastiere 2008). Because oral malignancy surgery involves important vascular and nerve tissues in the head and neck, prolonged anaesthesia, surgery, postoperative intensive care unit (ICU) stay, and hospitalisation are required. It is more likely to cause POCD in elderly patients. This study explored and analysed the incidence of POCD and its associated risk factors in elderly patients after expanded resection of primary oral malignancy and cervical lymphatic dissection.

## Materials and methods

### Patient selection and grouping

We selected 164 patients who underwent oral malignant tumour resection and cervical lymphatic dissection through general anaesthesia between December 2020 and December 2021. The inclusion criteria were as follows: American Society of Anesthesiologists (ASA) I–III, age ≥ 55 years, education level to complete the cognitive function measurement, and willingness to cooperate. The exclusion criteria were as follows: a mini-mental state examination (MMSE) score ≤ 23 points; central nervous system disease or cerebrovascular disease with sequelae; sedative and anti-depressants, severe alcoholism, or drug dependence; auditory or visual disorders; psychiatric disorders, posttraumatic stress disorder, developmental or mental intellectual disability, and cognitive impairment; previous seizures; history of cardiac surgery and neurosurgery; and previous neuropsychological tests.

During the course of the experiment, some patients abandoned surgery for reasons, or changed the operation type, or were taken intraoperative tracheotomy, or were reluctant to complete the postoperative evaluation. Finally, 112 subjects were divided into the POCD and non-POCD groups according to whether POCD occurred within 7 postoperative days. The study was performed with the approval of the Ethics Committee of the Stomatological Hospital affiliated with Chongqing Medical University.

### Anaesthesia method

Several studies have reported a correlation between the depth of anaesthesia and drugs in POCD (Ishii et al. 2016; Tang et al. 2014). Therefore, a unified anaesthesia protocol was used in this study.

All patients underwent intravenous-inhalation combined anaesthesia, induced with midazolam (0.04 mg/kg), rocuronium (0.6 mg/kg), remifentanil (2 ug/kg), and 1% propofol (1.5–2 mg/kg). After nasal tracheal intubation, anaesthesia machine was connected, using capacity control mode. After induction, internal body temperature was monitored, and internal jugular vein and dorsal foot artery were catheterised to value the central venous pressure and arterial blood pressure to maintain circulation stability. Anaesthesia was maintained by pumping propofol for sedation [(3–5 mg/(kg·h)], remifentanil for analgesia [0.05–0.15 g/(kg·min)], and inhaling sevoflurane 1.5–2%; muscle relaxation was maintained by rocuronium. EtCO_2_ and inhale concentration were monitored, and the BIS value was between 50 and 60. After operation, patients were connected to the analgesic pump and sent to ICU. The patient-controlled intravenous analgesia parameter was as follows: sufentanil 1 ug/kg + dezocine 0.15 mg/kg + dexmedetomidine 1 ug/kg + 0.9% saline to 100 mL, load 2 mL, continuous injection 2 mL/h, amount of automatic analgesia 1 mL, and locking time 15 min.

### Recorded indicators

Possible related risk factors for POCD were recorded, including general preoperative conditions: gender, age, body mass index (BMI), education level, ASA grade, smoking and alcohol consumption history, preoperative serum potassium concentration, history of surgery under general anaesthesia, preoperative disease, dyssomnia, and preoperative cognitive function. Surgery-related conditions include duration of operation, intraoperative bleeding volume, intraoperative hypotension (dorsal foot arterial systolic pressure is 20% lower than that before anaesthesia), and postoperative conditions, including duration of ICU stay, infection, second operation within 7 days after the first one, and postoperative cognitive function.

### Test tools

As accurate language test cannot be performed in patients who were taken oral malignancy surgery within 7 postoperative days, the cognitive function at the 1st and 3rd postoperative day could not be evaluated.

All patient tests were performed by dedicated experimenters between 2 and 5 p.m. Sleep disorders were assessed using the Pittsburgh Sleep Quality Index scale, which had a total score of 21 points with seven components, with a maximum of 3 points per component. Higher scores indicate greater sleep disturbances (Buysse et al. 1989).

Cognitive function assessment was performed the day before operation and the 7th postoperative day using multiple neuropsychological test scales to evaluate patient cognitive function, including the following:

1. MMSE: It is the most widely used cognitive function assessment scale, which includes time and location orientation, immediate language memory, attention, and computing, with the highest score of 30 points. However, it has a low sensitivity to mild cognitive impairment (MCI) (Mitchell 2009). In recent years, its applications have been limited due to copyright issues and poor sensitivity (Tsoi et al. 2015).
2. Addenbrooke’s cognitive examination (ACE-III), with a maximum score of 100, takes 15–20 min and includes the assessment of five cognitive domain, total 21 cognitive tests. Hsieh et al. (2013) found that subtests of attention, memory, language, and optic spatial ability in ACE-III were consistent with standard neuropsychological tests; studies have shown that when the cut-off value is 85 points, the sensitivity of the Chinese version of ACE-III diagnosis is 97.3%, and the specificity is 90.7% (Wang et al. 2019).
3. Memory and execution screening (MES): It is mainly used for screening for MCI, without subject writing, little education impact, and high sensitivity and specificity (Guo et al. 2012). The highest score is 100 points; it takes 5–10 min, which can assess memory and execution comprehensively. Guo et al. (2012) found that the MES total score area under the curve (AUC) (95% confidence interval [CI], 0.85–0.92) was 0.89, with a sensitivity of 79.5% and a specificity of 82.8%. When the cut-off value was set to < 75, there was a correct classification rate of 81%; the MES total score AUC (95% *CI*, 0.93–0.97) was 0.95, the sensitivity was 87%, the specificity was 91%, and when the cut-off value was set to 72, the correct classification rate was 90%.

For those who were provided informed consent but were unwilling to undergo surgery, 15 patients were selected as controls to undergo a cognitive function test. The diagnosis of POCD was made using the “Z-score” comprehensive score method recommended by the International Study of POCD (Moller et al. 1998). POCD was diagnosed if two or more scale tests of *Z*-value ≥ 2 or a total *Z*-value of ≥ 2 existed in the same patient, eliminating the learning effect in the experimental group; this is a more reasonable method for the diagnosis of POCD (Hanning 2005).

### Statistical analysis

Statistical analysis was performed using SPSS 26.0 software package. Normal test and homogeneity of variance test were performed separately; normal distribution measures are presented as *x* ± *s*, using two independent-sample *t*-tests or nonparametric anecdotal sum tests (Wilcoxon tests) between groups, and skewed distribution measurements are presented as a median and interquartile range, using Mann–Whitney *U*-test for group comparisons, and within-group differences were determined by one-way analysis of variance. Count data were statistically analysed using a chi-square test or Fisher exact probability method, and multivariate analysis was performed using logistic regression analysis (*P* < 0.05).

## Results

### The general condition of the patient

**Fig. 1:**
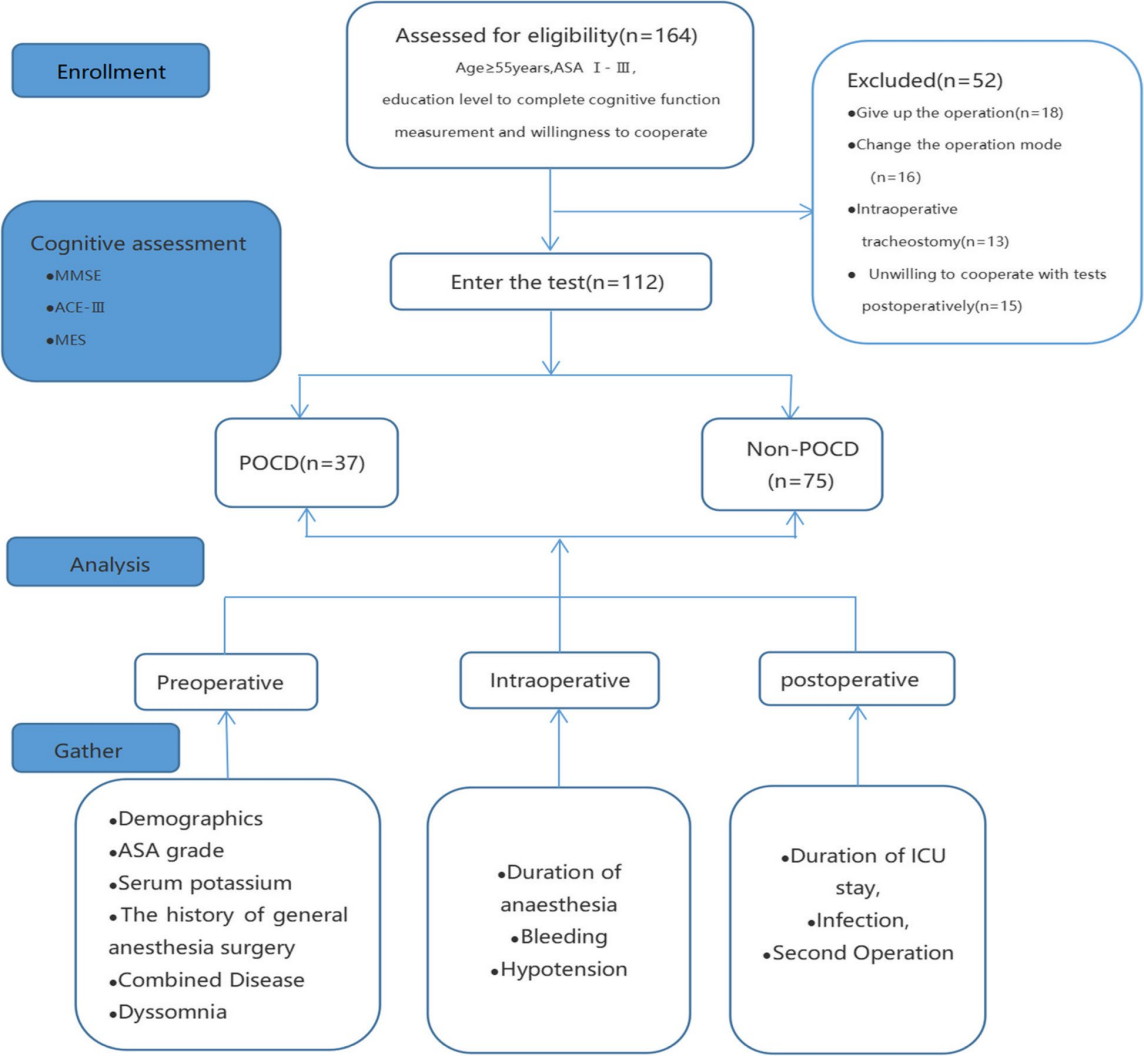
Research design flow chart. Note: Cognitive function assessments were performed 1 day preoperatively and 7 days postoperatively

A total of 164 patients were included in the trial; finally, 112 patients completed the study. Figure 1 illustrates the study flow chart. There were 72 men (64.3%) and 40 women (35.7%), and the average age was 63.83 ± 3.43 years. Judging by the “Z-score” comprehensive scoring method, the incidence of POCD within 7 days was 33.1%, including 37 and 75 patients in the POCD and non-POCD groups, respectively.

The results of the cognitive function tests in both groups are shown in Table 1; the day 7 postoperative test results in the POCD group were worse than those of the non-POCD group, and MMSE, ACE-III, and MES scores were significantly different compared to the non-POCD group (*P* < 0.05). The ACE-III and MES scores were significantly different in the POCD group compared to the preoperative scores on postoperative day 7 (*P* < 0.05).

**Table 1:** Neuropsychological test results of the POCD and non-POCD groups (x ± s)

	POCD (*n* = 37)		Non-POCD (*n* = 75)	
	1 day before surgery	7 days after surgery	1 day before surgery	7 days after surgery
MMSE	25.86 ± 1.53	23.05 ± 1.68*	25.55 ± 1.38	25.56 ± 1.46
ACE-III	73.73 ± 8.17	65.89 ± 7.71#*	72.85 ± 6.09	72.69 ± 6.17
MES	72.24 ± 9.60	62.38 ± 9.17#*	70.09 ± 5.59	69.8 ± 5.28

*POCD* Postoperative cognitive dysfunction, *non-POCD* No postoperative cognitive dysfunction, *MMSE* Mini-mental state examination, *ACE-III* Addenbrooke’s cognitive examination, *MES* Memory and execution screening

Compared to the same group, # indicates *P* < 0.05; compared to the non-POCD group preoperative, * indicates *P* < 0.05

### Comparison of general physical signs and preoperative indicators

A comparison of the general preoperative characteristics and perioperative indicators from 2 group patients is shown in Table 2. Univariate analysis revealed that venerable age, low education levels, preoperative history (including diabetes, hypertension, coronary heart disease, and cerebral infarction), reduced serum potassium concentration, and dyssomnia can increase the risk of POCD within 7 days postoperatively. Patients in the POCD group were elderly and had a higher incidence of diabetes, hypertension, coronary heart disease, cerebral infarction, low potassium levels, and sleep disturbance. Meanwhile, there were no significant differences between the POCD and non-POCD groups regarding sex, BMI, ASA status, living habits, and respiratory history (*P* > 0.05).

**Table 2:** Demographics and clinical data associated with POCD in elderly patients

	POCD (*n* = 37)		Non-POCD (*n* = 75)		*P*-value
Age (year)	65.03 ± 3.69		63.24 ± 3.16		0.023
Sex (*n*, %)					0.742
Women	14	37.80%	26	34.70%	
Men	23	62.20%	49	65.30%	
BMI (kg m^−2^)	24.58 ± 1.60		24.36 ± 1.12		0.456
ASA grade (*n*, %)					0.383
I	15 (40.50%)		36 (48%)		
II	18 (48.60%)		34 (45.30%)		
III	4 (10.80%)		5 (6.70%)		
Education level (years)	4.97 ± 2.65		6.31 ± 2.61		0.017
Living habits (*n*, %)					
History of smoking	14 (37.80%)		33 (44.00%)		0.534
History of drinking	15 (40.50%)		31 (41.30%)		0.936
Medical history (*n*, %)					
History of general anaesthesia surgery	12 (32.40%)		23 (30.70%)		0.850
Diabetes mellitus	9 (24.30%)		6 (8.00%)		0.035
Hypertension	16 (43.20%)		14 (18.70%)		0.006
Coronary disease	8 (21.60%)		5 (6.70%)		0.029
Disease of the respiratory system	3 (8.10%)		3 (4.00%)		0.364
History of cerebral infarction	7 (18.90%)		4 (5.30%)		0.039
Sleep disorders (points)	5.51 ± 2.70		4.03 ± 1.99		0.010
Low potassium	9 (24.30%)		7 (9.30%)		0.033

*POCD* Postoperative cognitive dysfunction, *non-POCD* No postoperative cognitive dysfunction, *BMI* Body mass index, *ASA* American Society of Anesthesiologists

### Comparison of perioperative characteristics

The univariate analysis results of the main perioperative characteristics are shown in Table 3. There were no significant differences between the two groups regarding intraoperative bleeding, duration of ICU stay, second-time surgery within 7 days, and postoperative infection (*P* > 0.05). However, prolonged anaesthesia and intraoperative hypotension can increase the incidence of POCD 7 days postoperatively (*P* < 0.05).

**Table 3:** Main perioperative factors associated with POCD in elderly patients

	POCD (*n* = 37)	Non-POCD (*n* = 75)	*P*-value
Time of anaesthesia (min)	514.05 ± 66.35	483.27 ± 61.77	0.013
Intraoperative hypotension (*n*, %)	9 (24.30%)	6 (8.00%)	0.035
Intraoperative bleeding (mL)	532.43 ± 127.59	566.67 ± 144.34	0.223
Length of ICU stay (days)	3 (2 ~ 4)	2 (2 ~ 3)	0.114
Secondary operation within 7 days (*n*, %)	5 (13.50%)	7 (9.30%)	0.527
Postoperative infection (*n*, %)	4 (10.80%)	7 (9.30%)	0.523

*POCD* Postoperative cognitive dysfunction, *non-POCD* No postoperative cognitive dysfunction, *ICU* Intensive care unit

### Analysis of the high-risk factors for POCD

Several associated risk factors with significant differences in the univariate analysis were subjected to binary logistic regression analysis. The results are presented in Table 4. Venerable age (odds ratio [OR] = 1.269, 95% CI 1.056–1.525, *P* < 0.05), low education levels (OR = 0.792, 95% CI 0.644–0.974, *P* < 0.05), hypertension (OR = 4.153, 95% CI 1.335–12.732, *P* < 0.05), sleep disorder (OR = 172, 95% CI 1.001–117, *P* < 0.05), prolonged anaesthesia (OR = 1.009, 95% CI 1.001–1.018, *P* < 0.05), and intraoperative hypotension (OR = 5.512, 95% CI 1.240–24.506, *P* < 0.05) were independent risk factors for POCD in elderly patients with oral malignancies.

**Table 4:** Multivariate logistic regression analysis of risk factors for POCD

	OR	95% CI		*P*-value
		Lower limit	Superior limit	
Venerable age	1.269	1.056	1.525	0.011
Low education level	0.792	0.644	0.974	0.027
Diabetes mellitus	2.924	0.653	13.093	0.161
Hypertension	4.153	1.355	12.732	0.013
Coronary disease	2.321	0.417	12.925	0.336
A history of cerebral infarction	3.543	0.639	19.649	0.148
Dyssomnia	1.272	1.001	1.617	0.049
Low potassium	1.206	0.240	6.066	0.820
Prolonged anaesthesia	1.009	1.001	1.018	0.035
Intraoperative hypotension	5.512	1.24	24.506	0.025

## Discussion

**Fig. 2:**
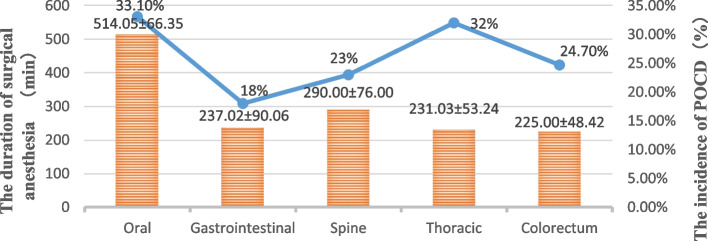
Comparison of the incidence of POCD with the duration of surgical anaesthesia in several common major surgeries. Note: Data were obtained from references (Yuan et al. 2019; Jiao et al. 2021; Li et al. 2021; Kim et al. 2016)

The incidence of POCD has been widely reported, with studies showing an incidence of 18–32% within 1 week after undergoing general anaesthesia and noncardiac surgery in elderly patients (Moller et al. 1998; Yuan et al. 2019; Jiao et al. 2021; Li et al. 2021; Kim et al. 2016). In the results of this study (Fig. 2), it was found that the duration of surgical anaesthesia was approximately twice that of other common major surgeries. The incidence of POCD at 1 week in elderly patients with oral malignancies was 33.1%, which is higher than that observed in other types of noncardiac surgery. This was possibly because of surgery for oral malignancy requiring tissue repair and reconstruction of the defect after tumour resection and extensive surgical traumas. And excessive stretching and rotation in cervical lymphatic surgery can cause intimal injury to the arterial lining of head and neck, thereby promoting the formation of microembolism, creating carotid artery stenosis, cerebral tissue hypoperfusion, and other risks to cause cerebral ischemia (Evered et al. 2011; Leiendecker et al. 2010). These factors often lead to brain damage in patients, causing a decline in cognitive function.

Venerable age is currently a more explicit risk factor for POCD, and similar results were obtained in this study. Several studies suggest that the pathogenesis of POCD is neuroinflammation, and it is age related (Luo et al. 2019). The mechanism may be a decrease in brain volume and white matter integrity with increasing age. Moreover, cerebral blood flow decreases with age, resulting in reduced oxygen delivery, slow metabolism, and age-induced central nervous system apoptosis, which affects neurons, synapses, and neurotransmitters, leading to an increased incidence of POCD in elderly patients. At the same time, elderly patients often combined with a variety of diseases, and the ability to cope with the injuries caused by surgery and anaesthesia is reduced. Therefore, the risk of perioperative complications and POCD also increases accordingly (Otomo et al. 2013). In conclusion, elderly patients are more likely to develop POCD after surgery.

The level of education is closely related to the occurrence of POCD (Huang et al. 2020). Feinkoh et al. (2017) found that in middle-aged and elderly patients undergoing surgery, years of education are inversely proportional to the incidence of cognitive impairment. Patients with high cognitive reserve display more brain activity and can better adjust or activate synaptic connections between neurons using neuronal reserve, bypassing damaged areas and increasing synaptic efficacy to deal with injury. In addition, with low education, they have more harmful factors in their living environment and a more unhealthier lifestyle. They may present more severe brain pathological manifestations than their peers, such as amyloid β-protein deposition, which exacerbated the cognitive deficiency by anaesthesia (Evered et al. 2016).

In recent years, studies have focused on the association between dyssomnia and POCD. The results obtained in this study showed that sleep disturbance is an independent risk factor for POCD. Sleep accounts for approximately one-third of an individual’s lifespan and is closely related to human health. Various aspects of sleep are affected in varying degrees in most elderly adults (Kang et al. 2017). Dyssomnia is not conducive to protein synthesis and establishment of new synaptic connections in the brain, affecting the cognitive change in cerebral cortical, leading to neuroendocrine disorders, decreased immune function, deterioration of behaviour, anxiety, depression, irritability, and other complications, thereby inducing or aggravating POCD (Gogenur et al. 2008). A meta-analysis revealed that various types of sleep disorders, such as difficulty remaining asleep, reduced sleep duration, reduced sleep efficiency, and daytime dysfunction, can significantly increase the risk of cognitive impairment (Bubu et al. 2017). Studies have demonstrated that the intraoperative use of dexmedetomidine can significantly stabilise patients’ haemodynamics, reduce the occurrence of inflammatory reactions, inhibit free radical generation, and reduce sleep disorders caused by the use of other anaesthetic drugs and has a certain protective effect on the sleep of patients under general anaesthesia (Guldenmund et al. 2017).

Hypertension is a common cardiovascular complication in elderly patients, and studies suggest that hypertension is often accompanied by cerebrovascular and carotid atherosclerotic plaque, which leads to cerebrovascular autoregulation function. Under the stimulation of various related factors during the perioperative period, there can be local or whole cerebral blood flow, cerebral oxygen content further declines, central nervous system transmitter release reduced, and particularly, the cholinergic nervous system function declined, leading to impaired brain function, making hypertensive patients more prone to postoperative cognitive function damage (Shaw et al. 2003). Spence et al. (2004) demonstrated that for every 10-mmHg increase in systolic BP, the risk of cognitive dysfunction increased by 7% compared with that of the control group. The systolic BP was > 160 mmHg, and the cognitive decline was significantly increased.

The duration of operation was also an independent risk factor for POCD onset in this group, and the mean duration of operation of patients in the POCD group was higher than that in the non-POCD group, and perhaps the more complex surgical steps led to the occurrence of POCD. More complex surgical steps imply a prolonged surgical anaesthesia duration. A previous study revealed a significant increased incidence of POCD in patients with surgery longer than 450 min, and a long-time surgery is a further important predictor of POCD (Otomo et al. 2020). A systematic review by Freddi Segal-Gidan et al. (2017) concluded that a shorter duration of operation was associated with a less risk of POCD. Animal studies confirmed that anaesthesia exposure can change amyloid and tau protein in mice, which leads to cognitive dysfunction (Segal-Gidan 2017).

Currently, the relationship between intraoperative blood pressure and postoperative cognitive function is controversial. Intraoperative hypotension in this study was an independent risk factor for POCD. Hypotension leads to a low perfusion state of the brain blood supply and induces free radical damage and other pathological changes. In addition, oxidative stress response can cause various changes in neuronal degeneration and protein apoptotic genes, reducing the molecular expression that creates and maintains synaptic connections, thereby impacting memory and cognitive function. A prospective clinical study demonstrated that a single longest cerebrovascular modulation of BP change over 5.03 min was associated with decreased postoperative cognitive function (Kumpaitiene et al. 2019). Therefore, sustained hypotension during general anaesthesia may cause damage to the nervous system of patients with chronic insufficient cerebral perfusion (such as elderly patients), and preventing intraoperative hypotension helps to prevent POCD (Yamamoto et al. 2018).

Due to the oral surgery, language testing could not be performed. To mitigate the impact on neuropsychology, the cognitive function of patients could not be studied within 7 postoperative days. In the multivariate analysis, some insensitive indicators were not analysed. In this study, diabetes, coronary disease, cerebral infarction, and hypokalaemia were associated with POCD but were not independent risk factors for POCD. Few studies have found postoperative cognitive impairment risk factors may also include intraoperative bleeding, prolonged ICU stay, second-time surgery, and postoperative infection. The above two conditions may be related to the present study. This study was a single-centre experiment with small sample size; therefore, the results showed no significant statistical differences. Consequently, multicentre observational studies involving large cohorts are required to determine whether current risk factors have high predictive value.

“Perioperative Brain Health Initiative”, which aims to focus on the anaesthesia-related brain health of elderly patients, explores effective perioperative brain protection measures and reduces the incidence of central nervous system complications, such as POCD in elderly patients. Therefore, the active prevention of POCD in elderly patients with oral malignancies is a problem that clinicians should pay significant attention to and resolve. In this study, the incidence of POCD 1 week after oral malignancy surgeries of the elderly patients was higher than that in other noncardiac surgeries. Venerable age, low education level, hypertension, sleep disturbance, long-time surgery, and intraoperative hypotension were independent risk factors for POCD in elderly patients with oral malignancies. Clinicians should understand the solutions to reduce the incidence of POCD, identify and manage the perioperative risk factors early, and provide effective preventive interventions and treatments for high-risk groups.

## Data Availability

All data generated or analysed during this study are not publicly available due to privacy but are available from the corresponding author upon reasonable request.
